# Environmental Viscosity Modulates Interbacterial Killing during Habitat Transition

**DOI:** 10.1128/mBio.03060-19

**Published:** 2020-02-04

**Authors:** Lauren Speare, Stephanie Smith, Fernanda Salvato, Manuel Kleiner, Alecia N. Septer

**Affiliations:** a Department of Marine Sciences, University of North Carolina, Chapel Hill, North Carolina, USA; b Department of Plant and Microbial Biology, North Carolina State University, Raleigh, North Carolina, USA; Imperial College London; University of Washington

**Keywords:** *Aliivibrio fischeri*, aggregation, competition, proteomics, type VI secretion

## Abstract

Symbiotic bacteria use diverse strategies to compete for host colonization sites. However, little is known about the environmental cues that modulate interbacterial competition as they transition between free-living and host-associated lifestyles. We used the mutualistic relationship between Eupyrmna scolopes squid and Vibrio fischeri bacteria to investigate how intraspecific competition is regulated as symbionts move from the seawater to a host-like environment. We recently reported that V. fischeri uses a type VI secretion system (T6SS) for intraspecific competition during host colonization. Here, we investigated how environmental viscosity impacts T6SS-mediated competition by using a liquid hydrogel medium that mimics the viscous host environment. Our data demonstrate that although the T6SS is functionally inactive when cells are grown under low-viscosity liquid conditions similar to those found in seawater, exposure to a host-like high-viscosity hydrogel enhances T6SS expression and sheath formation, activates T6SS-mediated killing in as little as 30 min, and promotes the coaggregation of competing genotypes. Finally, the use of mass spectrometry-based proteomics revealed insights into how cells may prepare for T6SS competition during this habitat transition. These findings, which establish the use of a new hydrogel culture condition for studying T6SS interactions, indicate that V. fischeri rapidly responds to the physical environment to activate the competitive mechanisms used during host colonization.

## Abstract (precis)

Bacteria often engage in interference competition to gain access to an ecological niche, such as a host. However, little is known about how the physical environment experienced by free-living or host-associated bacteria influences such competition. We used the bioluminescent squid symbiont Vibrio fischeri to study how environmental viscosity impacts bacterial competition. Our results suggest that upon transition from a planktonic environment to a host-like environment, V. fischeri cells activate their type VI secretion system, a contact-dependent interbacterial nanoweapon, to eliminate natural competitors. This work shows that competitor cells form aggregates under host-like conditions, thereby facilitating the contact required for killing, and reveals how V. fischeri regulates a key competitive mechanism in response to the physical environment.

## INTRODUCTION

The genomes of many host-associated bacteria encode diverse strategies for interbacterial competition, including the type VI secretion system (T6SS) (1–6), which is a contact-dependent killing mechanism. This protein export machinery has been identified in commensals (2, 7–9), pathogens (1, 3–6, 10, 11), and beneficial symbionts (2, 7) and is predicted to critically impact bacterial fitness in the host (12–16). The T6SS employs a toxin/immunity system to kill or inhibit competitor cells: an inhibitor cell builds a proteinaceous tube that acts as a molecular syringe to deliver toxic effector proteins directly into target cells, resulting in growth inhibition or cell death if the target lacks the appropriate immunity proteins (16–18). The T6SS structure, T6SS assembly, and classes of T6SS effectors have been elucidated by using isogenic mutants of several model organisms, including Vibrio cholerae and Pseudomonas aeruginosa (1, 3, 16, 19). More recently, researchers have turned to coculture experiments with nonisogenic strains to investigate the potential for T6SS interactions to influence the composition of natural microbial communities (2, 19–22).

Recent evidence suggests that T6SSs are active in animal microbiomes (2–6, 12, 13, 20, 22–24) and may be modulated by host-specific biochemical cues (4, 25). For example, mucin proteins promote the T6SS function of pandemic V. cholerae strains (25), and both V. cholerae and Salmonella enterica enhance T6SS function in the presence of bile salts (4, 25). Moreover, the transcription of one T6SS in P. aeruginosa is induced under iron-limiting conditions (26), which are found within the host environment. Despite the prevalence of T6SSs in host-associated bacteria, few studies have investigated how the host’s physical environment impacts T6SS function (25).

Like many horizontally acquired bacteria, the beneficial squid symbiont Vibrio fischeri experiences dramatic changes in its environment as it transitions between a free-living lifestyle in an aquatic habitat to a host-associated lifestyle. Juvenile squid hatch without their bacterial symbiont, which they acquire from the surrounding seawater (27). We previously showed that the V. fischeri genome encodes a strain-specific T6SS on chromosome II (T6SS2) that is used for interstrain competition during the initial colonization of the squid host (2). T6SS2 is necessary for one strain type (inhibitor) to prevent another strain type (target) from occupying the same colonization site. Because this T6SS provides strains with an advantage by excluding other genotypes as they compete for a limited number of sites in the host, we became interested in determining how the T6SS2 function is modulated in response to changes in the physical environment upon the transition from seawater to the host.

Although there are many differences between the chemical and physical properties of seawater and those of the host, here we focused on one factor: viscosity. During colonization, free-living V. fischeri transitions from a lower-viscosity aquatic habitat in the water column to a higher-viscosity environment as it becomes entrained in the host-generated fluid flow that facilitates the transport of bacterial cells toward the light organ surface (28), which is covered in mucus (29, 30). Here, symbiotic cells form and remain in aggregates for ∼15 to 180 min, depending on the strain type (31), before transiting to crypt spaces within the light organ, which contain a highly viscous lumen (32, 33). Moreover, some cells interact directly with the surface of host epithelial cells lining the crypts (34). Therefore, we predicted that differences in environmental viscosity upon habitat transition may be perceived by potential colonizers to be a host-specific cue to modulate T6SS-mediated competition.

In this study, we leveraged the ability to examine interstrain competition *in vitro* using V. fischeri isolates that naturally compete for the same niche to determine how cells regulate T6SS2 in response to a change in viscosity and surface association. We examined T6SS2 modulation at three levels: transcription of essential T6SS genes, assembly of the molecular syringe, and contact with target cells. In addition, we explored the capacity of a short exposure time under high-viscosity (mucus-like) conditions to prime cells for T6SS2-mediated killing. Finally, we used proteomics to determine the global cellular response to transitioning from a low-viscosity environment to a high-viscosity environment. This study illustrates how symbionts can rapidly respond to their physical environment as they prepare to engage with competitors of a host niche.

## RESULTS AND DISCUSSION

### PVP can control environmental viscosity in culture.

Although the surface-based coincubation assays described previously were essential for identifying and characterizing T6SS2 function in V. fischeri (2), they are not suitable to replicate key changes in the physical environment that symbionts experience when moving from aquatic to host habitats. Therefore, we sought to manipulate the viscosity of the liquid medium for use in our *in vitro* competition assay to better reflect the physical environments encountered by V. fischeri during habitat transition.

Previous studies have successfully used the water-soluble polymer polyvinylpyrrolidone (PVP) to increase the viscosity of bacterial culture media. When PVP is added to the medium at 2.5% to 10% by weight per volume (wt/vol), a high-viscosity hydrogel results (35–37). We first determined how PVP-amended medium impacts the growth of our V. fischeri strains. We grew strain ES114 or ES401 alone in minimal medium (MM) either with no added carbon source or supplemented with 10 mM *N*-acetylglucosamine (NAG), 5% (wt/vol) PVP, or a combination of 10 mM NAG and 5% PVP. Medium supplemented with 5% PVP was visibly more viscous and resulted in a hydrogel with a viscosity of ∼152 centipoise (cP). For reference, the viscosity of water at room temperature is ∼1 cP (38). After 12 h, both strains reached similar cell densities when grown in MM supplemented with NAG alone or NAG and PVP but were unable to grow in MM with no carbon source or when PVP was the sole carbon source (see Fig. S1 in the supplemental material). These data demonstrate that (i) the V. fischeri strains tested here are unable to use PVP as a carbon source and (ii) PVP does not impact the growth yield, at least within the first 12 h. Therefore, we concluded that supplementation of the medium with PVP is a good approach for controlling viscosity in liquid coincubation assays.

10.1128/mBio.03060-19.2 FIG S1 V. fischeri does not use PVP as a sole carbon source. Growth yields of clonal cultures of strains ES114 (cyan) and ES401 (magenta) grown for 12 h in minimal medium supplemented with 10 mM *N*-acetylglucosamine (NAG) or 5% PVP. Experiments were performed three times, and combined data are shown (*n* = 12). Treatments that do not share letters denote a statistically significant difference (Student’s *t* test, *P < *0.001). Download FIG S1, DOCX file, 0.1 MB. Copyright © 2020 Speare et al. 2020 Speare et al. This content is distributed under the terms of the Creative Commons Attribution 4.0 International license.

### High-viscosity liquid promotes T6SS2-dependent competition.

**FIG 1:**
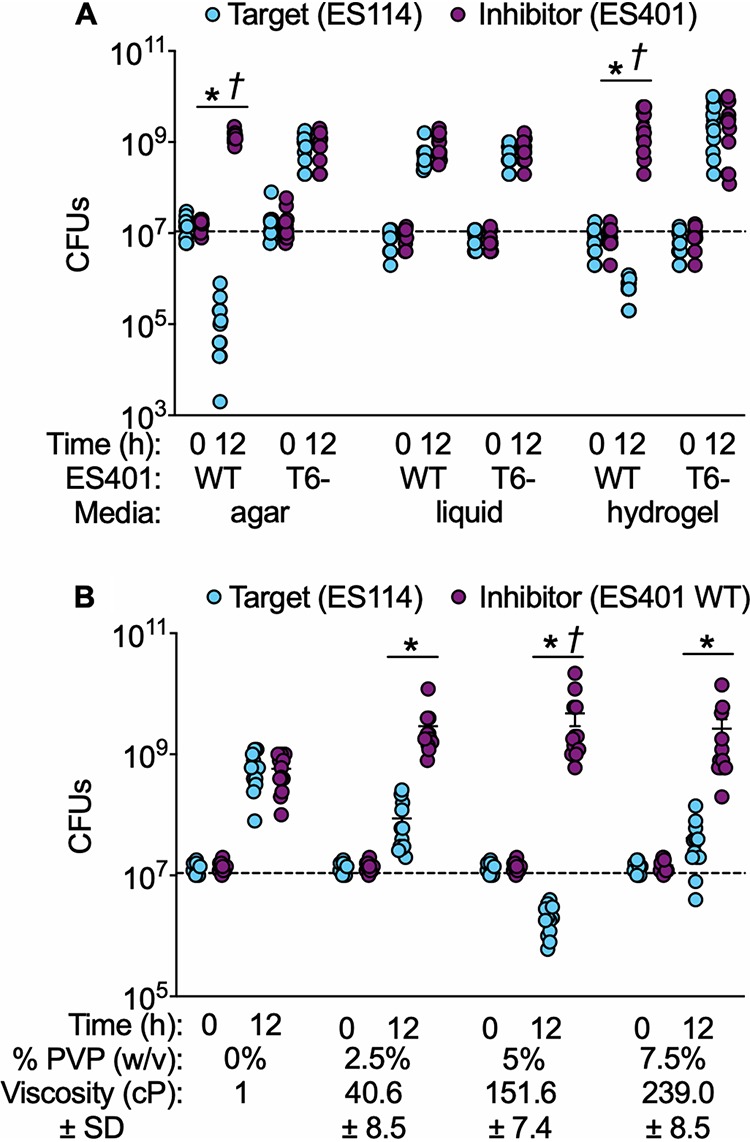
ES401 kills ES114 in a T6SS2-dependent manner on agar surfaces and in high-viscosity liquid medium. CFUs of ES114 (cyan) and wild-type (WT) or *vasA_2* mutant (T6SS2 mutant [T6^−^]) ES401 (magenta) were determined in coincubation assays performed on agar surfaces (agar) or in low- (liquid) or high-viscosity (hydrogel) liquid medium (A) or in media with a range of viscosities (B). Dashed lines indicate the average number of CFUs at 0 h. The viscosity of liquid medium without PVP (0%) is based on the viscosity of water. Asterisks indicate that ES401 had statistically significantly higher CFUs than ES114 at 12 h (Student's *t* test, *P < *0.03), and daggers indicate that the CFUs for ES114 statistically significantly decreased from 0 h to 12 h (Student's *t* test, *P < *0.0001). Error bars indicate SEMs. Experiments were performed at least three times, and combined data are shown (*n* = 12).

We next used coincubation assays to quantify T6SS2-dependent interactions between two differentially tagged V. fischeri light organ isolates. We chose ES114 as the target strain (39) because its genome does not encode the T6SS2 genomic island (2) and ES401 as the inhibitor strain (27) because its genome contains the T6SS2 genomic island (40) and kills ES114 in coculture experiments on agar surfaces (2). When ES114 was coincubated with wild-type ES401 on agar surfaces, the number of colony-forming units (CFUs) for ES114 decreased significantly from the starting number of CFUs and were significantly lower than the ES401 CFUs at 12 h (Fig. 1A). In contrast, when ES114 was coincubated with the ES401 *vasA_2* (*tssF*) mutant strain, which lacks the baseplate component required for T6SS function (2), the CFUs for both strains increased after 12 h and were not significantly different from each other (Fig. 1A). These findings indicate that ES401 outcompetes ES114 on agar surfaces using T6SS2, which is consistent with previous findings using other T6SS2-positive (T6SS2^+^) strains (2). When coincubation assays were performed in low-viscosity liquid, CFUs for ES114 increased after 12 h in the presence of both the wild-type ES401 and *vasA_2* mutant strains (Fig. 1A). In contrast, coincubation assays in high-viscosity liquid showed ES114 CFUs significantly decreased after 12 h and were significantly lower than the CFUs for wild-type ES401, while CFUs of both strains increased when ES114 was incubated with the *vasA_2* mutant (Fig. 1A). These data indicate that ES401 can outcompete a target strain in a T6SS2-dependent manner when strains are coincubated in a hydrogel medium.

Given that the viscosities of the juvenile light organ mucus and crypt lumen have not yet been directly quantified, we wanted to determine whether ES401 outcompeted ES114 across a range of viscosities. We next performed coincubation assays in media with viscosities ranging from 1 cP to 239 cP (Fig. 1B). The CFUs for ES114 were significantly lower than the CFUs for ES401 in all PVP-amended treatments (Fig. 1B). Importantly, the largest competitive effect was observed at 152 cP (5% PVP), which is within the range of viscosities observed for mucus in other hosts where sufficient samples can be obtained for direct measurement (41). Taken together, these data suggest that T6SS2-dependent competition is modulated by the physical environment: ES401 kills a target strain on surfaces and in high-viscosity liquid but does not do so in low-viscosity liquid. We considered three hypotheses to explain how the T6SS2 function is modulated in response to viscosity/surfaces: (i) T6SS2 is transcriptionally regulated by viscosity, (ii) T6SS2 sheath assembly is regulated by viscosity, and/or (iii) high viscosity promotes cell-cell contact between strains.

### Viscosity regulates T6SS2 promoter activity and sheath formation.

**FIG 2:**
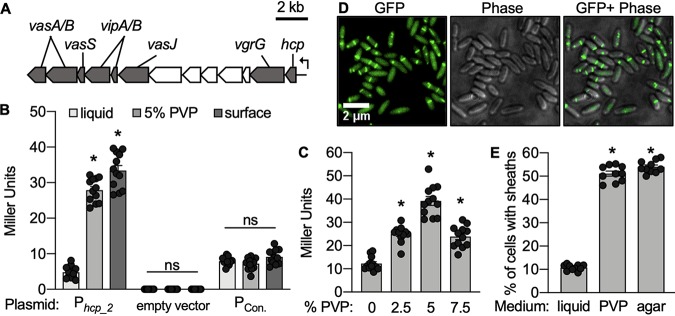
Viscosity regulates T6SS2 transcription and sheath formation. (A) Gene map for the predicted multigene operon (VFES401_RS15775 to VFES401_RS15830) (40) within the V. fischeri T6SS2 genomic island showing conserved T6SS genes (gray) and genes of unknown function (white). (B) β-Galactosidase assays were performed on cells grown on agar surfaces or in low-viscosity (liquid) or high-viscosity (5% PVP) medium. Treatments included ES401 harboring a T6SS2 reporter plasmid (pAG01), the empty vector (pAKD701), or a constitutive promoter (pJLB207). (C) β-Galactosidase assays were performed on ES401 cells harboring pAG01 grown in high-viscosity liquid. (B and C) *, *P < *0.0001 by a Student's *t* test comparing Miller unit values between treatments; ns, not significant (*P > *0.05). (D) Representative GFP images, phase-contrast images (Phase), and an overlay of GFP and phase-contrast images of ES401 cells expressing VipA_2-GFP after incubation in high-viscosity liquid for 2 h. (E) Percentage of VipA_2-GFP-expressing cells that contain sheaths after being incubated in low-viscosity liquid (liquid) or high-viscosity liquid (PVP) or on an agar surface (agar) for 2 h. All medium types were supplemented with 0.5 mM IPTG (isopropyl-β-d-thiogalactopyranoside) to induce expression of VipA_2-GFP. *, *P < *0.0001 by a Student's *t* test comparing the percentage of cells with sheaths in liquid to the percentage of cells with sheaths on hydrogel or agar surfaces. Error bars indicate standard errors. Experiments were performed two (E) to three (B and C) times, and combined data are shown (*n* = 12 for panels B and C and *n* = 20 for panel E).

To test our first hypothesis, whether a high-viscosity/surface environment activates T6SS2 gene expression, we constructed a *lacZ*-based promoter reporter for *hcp_2* (*tssD*), an essential T6SS structural gene (19, 42). The *hcp_2* promoter is encoded by a sequence upstream of a possible multigene operon that includes several essential T6SS structural genes (Fig. 2A), and therefore, serves as a good indicator of T6SS2 transcription. Plasmids containing the *hcp_2* reporter (P*_hcp_2_*), a promoterless *lacZ* (empty vector), or a constitutive promoter driving the expression of *lacZ* (P_Con_) (43) were moved into strain ES401. Promoter activity was determined for strains incubated in each medium type by performing β-galactosidase assays and calculating the number of Miller units as described previously (44). Promoter activity for P*_hcp_2_* was significantly higher in high-viscosity liquid and on agar surfaces than in low-viscosity liquid, while Miller unit values were not significantly different between medium types for the empty vector or the vector containing the constitutively active promoter (Fig. 2B). These data suggest that the increase in P*_hcp_2_* activity is not an artifact of cell growth in high-viscosity liquid or on surfaces but, rather, is due to the transcriptional regulation of T6SS2 gene expression in response to the physical environment. Consistent with our observation that T6SS2 is functionally active across a range of viscosities (Fig. 1B), *hcp_2* promoter activity was significantly enhanced in all PVP-amended media compared to that in the low-viscosity liquid (Fig. 2C). These data indicate that the *hcp_2* promoter is activated by surface and high-viscosity liquid conditions across a range of viscosities.

We next tested how the physical environment impacts T6SS2 sheath formation. Given that P*_hcp_2_* is activated upon the transition from low- to high-viscosity liquid and surfaces and that VipA-VipB (TssB-TssC) sheath components are encoded downstream of this promoter (40), we hypothesized that the proportion of cells with T6SS2 sheaths would increase upon the transition from low- to high-viscosity liquid/surfaces. To test this hypothesis, we used single-cell fluorescence microscopy to image cultures of ES401 harboring an inducible VipA_2-green fluorescent protein (GFP) expression vector that was previously used to visualize T6SS2 sheaths in T6SS2^+^ V. fischeri isolates (2). When ES401 cells expressing VipA_2-GFP were incubated in high-viscosity liquid, diffuse GFP was observed in all cells, indicating that the entire population expressed VipA_2-GFP, and many of these cells had VipA_2-GFP sheaths assembled (Fig. 2D). Moreover, the proportion of cells with sheaths grown to mid-exponential phase in low-viscosity liquid was ∼10%; however, when these cells were transitioned to 5% PVP or surfaces and incubated for 2 h, the proportion of cells with sheaths significantly increased to ∼50% (Fig. 2E). Together, these data suggest that moving from a low-viscosity environment to a high-viscosity or surface environment modulates the proportion of cells in a population that assemble T6SS2 sheaths.

### T6SS2 activation in high viscosity is independent of key quorum-sensing genes.

We considered the possibility that higher-viscosity medium may impact the quorum-sensing (QS) pathway, which regulates luminescence in V. fischeri and which has been shown to regulate T6SSs in other species (26, 45–47). To explore this possibility, we first determined the impact of cell density alone on T6SS2 activation by quantifying the proportion of cells with sheaths for wild-type cultures grown in low-viscosity medium. As the culture density increased, the proportion of cells with sheaths remained at ∼10% (Fig. S2A), suggesting that cell density alone does not impact T6SS2 sheath assembly. To directly test the possibility that QS pathways impact T6SS2 activity in high-viscosity medium, we generated *luxR* and *luxU* regulatory mutants of the inhibitor strain, strain ES401. LuxR regulates downstream genes in response to the QS molecules *N*-octanoyl-l-homoserine lactone C8-HSL and *N*-3-oxohexanoyl-homoserine lactone (3OC6-HSL) (48, 49), and LuxU responds to C8-HSL and autoinducer-2 (AI-2) (50, 51). We chose to disrupt key proteins that sense and respond to QS molecules, rather than the synthesis proteins themselves, because the target strain can still produce the QS molecules, which could complement such mutations in the ES401 inhibitor in coculture (52). To ensure that these regulatory proteins function in ES401 in high viscosity as expected, we grew wild-type and *vasA_2*, *luxR*, and *luxU* mutant strains in high-viscosity medium and monitored cell density and luminescence over time. Consistent with the known role for these genes (50, 53), luminescence values were lower for the *luxR* mutant and higher for the *luxU* mutant than for the wild type (Fig. S2B). To directly test a role for these QS proteins in T6SS2 function in hydrogel, we performed coincubation assays with each of these mutants in high-viscosity liquid. We found that the *luxR* and *luxU* mutants were able to kill target cells to the same extent as the wild type (Fig. S2C), indicating that activation of T6SS2 in high viscosity is independent of these QS genes.

10.1128/mBio.03060-19.3 FIG S2 T6SS2 activation is independent of cell density and key quorum-sensing genes. (A) Cultures of ES401 harboring VipA_2-GFP were grown in liquid Luria-Bertani with added salt (LBS) supplemented with 0.5 mM IPTG (isopropyl-β-d-thiogalactopyranoside) to an OD_600_ of 0.5 to 2.0 and imaged immediately. (B) Luminescence curve showing specific luminescence (relative light units [RLU]/OD_600_) for the ES401 wild type (WT), *vasA_2* mutant*, luxU* mutant, and *luxR* mutant strains in high-viscosity liquid medium (5% PVP). (C) Colony-forming units (CFUs) from a coincubation assay between the ES114 (cyan) and ES401 (magenta) strains that were incubated in high-viscosity liquid medium for 12 h. *, *P < *0.001 by a Student’s *t* test comparing the CFUs of ES114 with the CFUs of ES401 at 12 h. Error bars indicate standard errors. All experiments were performed either twice with two biological replicates and five fields of view for each replicate (A) or three times with four biological replicates (B and C). Combined data are shown (*n* = 20 for panel A and *n* = 12 for panels B and C). Download FIG S2, DOCX file, 0.2 MB. Copyright © 2020 Speare et al. 2020 Speare et al. This content is distributed under the terms of the Creative Commons Attribution 4.0 International license.

### Short exposure to high viscosity primes cells for T6SS2 killing.

**FIG 3:**
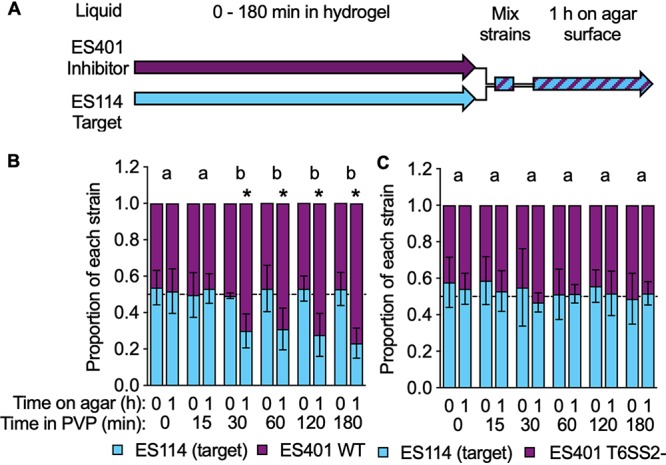
Exposure to high-viscosity liquid medium results in the earlier detection of T6SS2 killing on agar surfaces. (A) Timeline for the experiments whose results are shown in panels B and C. Liquid cultures of ES114 (cyan) and ES401 (magenta) were exposed to high-viscosity medium (liquid medium plus 5% [wt/vol] PVP) for 0 to 180 min, and then the strains were mixed in a 1:1 ratio and incubated on an agar surface for 1 h. CFUs were collected immediately (0 h) and after 1 h on the agar surface. Experiments were performed with ES114 and either wild-type ES401 (WT) (B) or *vasA_2* ES401 mutant (T6SS2 mutant [T6SS2^−^]) (C) ES401. Dashed lines indicate a proportion of 0.5. Letters indicate significantly different proportions of ES114 at 1 h on an agar surface between treatments (Student's *t* test, *P < *0.0001). *, *P < *0.0001 (Student's *t* test) comparing the proportion of ES114 to the proportion of ES401 at a given time point. Error bars indicate standard deviations. Experiments were performed three times, and the results of a representative experiment are shown (*n* = 4).

Given that ES401 cells respond to environmental viscosity by increasing sheath formation within 2 h, we hypothesized that T6SS2 killing is also activated within 2 h of exposure to high viscosity and is detectable at the population level. To test this hypothesis, we grew the target and inhibitor strains alone in low-viscosity liquid to an optical density at 600 nm (OD_600_) of ∼1.0 and then transferred these cells to high-viscosity liquid, where they remained for 0 to 180 min. Primed strains were then mixed at a 1:1 ratio and spotted onto an agar surface to force cells into physical contact to permit detection of T6SS2-mediated killing. CFUs were collected immediately (0 h) and after 1 h on the agar surfaces (1 h) (Fig. 3A). For all treatments, the initial proportion of ES114 to ES401 was not significantly different, indicating that the strains started the incubation on the agar surfaces at a 1:1 ratio (Fig. 3B). When strains were exposed to high-viscosity liquid for 0 or 15 min prior to mixing, ES114 and ES401 still maintained a 1:1 ratio after 1 h on the agar surfaces (Fig. 3B). However, when the strains were exposed to high-viscosity liquid for 30 to 180 min prior to mixing, ES114 comprised a significantly smaller proportion of the population after 1 h on the agar surfaces (Fig. 3B). This smaller proportion of ES114 in the coincubations after 1 h did not change with increasing exposure time to high-viscosity liquid medium, suggesting that more time in the hydrogel did not further enhance T6SS2 activation in ES401. When the same experiment was performed with ES114 and the ES401 *vasA_2* mutant, both strains maintained a 1:1 ratio throughout the experiment across all treatments (Fig. 3C). These data suggest that a 30-min exposure to a high-viscosity environment prior to physical contact with target cells is sufficient for ES401 to activate T6SS2 and outcompete ES114 in a T6SS2-dependent manner. These findings are consistent with those of past studies that found evidence for host-specific chemical signals priming V. fischeri for important cellular processes in the host (54–58). Thus, V. fischeri likely encounters both physical and biochemical cues to prime cells for its host-associated lifestyle.

### V. fischeri forms aggregates in high-viscosity liquid.

**FIG 4:**
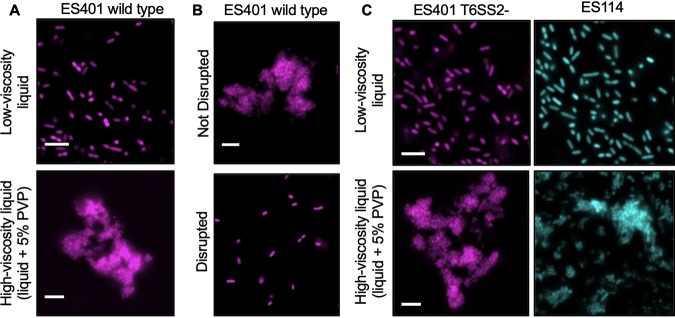
V. fischeri forms aggregates in high-viscosity, liquid medium. Fluorescence microscopy images of GFP-tagged ES114 (cyan) or RFP-tagged wild-type or *vasA_2* mutant (T6SS2 mutant) ES401 (magenta) incubated in the specified medium type for 12 h. (A) The ES401 wild type was incubated in either low-viscosity liquid or high-viscosity liquid. (B) The ES401 wild type was incubated in high-viscosity liquid and either spotted directly onto a glass slide (Not Disrupted) or mixed prior to spotting (Disrupted). (C) The ES401 *vasA_2* mutant and ES114 were incubated in either low-viscosity liquid or high-viscosity liquid. For all fluorescence microscopy images, each experiment was performed twice with two biological replicates and five fields of view. One representative image is shown. Bars = 5μm.

To test whether a high-viscosity environment promotes cell-cell contact, which is required for T6SS-mediated interactions, we first visualized a monoculture of fluorescently tagged ES401 grown in low- or high-viscosity liquid using single-cell microscopy. In low-viscosity liquid, cells were dispersed and very few neighboring cells were touching (Fig. 4A). In contrast, when ES401 was grown in high-viscosity liquid, the majority of cells formed multicellular aggregates (Fig. 4A). These aggregates could be easily disrupted by pipetting up and down (Fig. 4B), suggesting that the cells were loosely associated and that the act of pipetting and mixing these cultures during the dilution series was sufficient to disperse the cells to permit the accurate determination of CFU counts, which were comparable to those obtained for low-viscosity liquid, in which no aggregation was apparent. To determine whether aggregate formation is dependent on a functional T6SS2 or the presence of the T6SS2 genomic island, we imaged cultures of the ES401 *vasA_2* mutant or ES114, which lacks the T6SS2 genomic island, when grown in low- or high-viscosity liquid. For both strain types, aggregates were observed only in high-viscosity liquid cultures (Fig. 4C), indicating that neither a functional T6SS2 nor the T6SS2 genomic island is required for aggregate formation in high-viscosity liquid.

### Aggregates form within 6 h of the transition to high-viscosity conditions.

**FIG 5:**
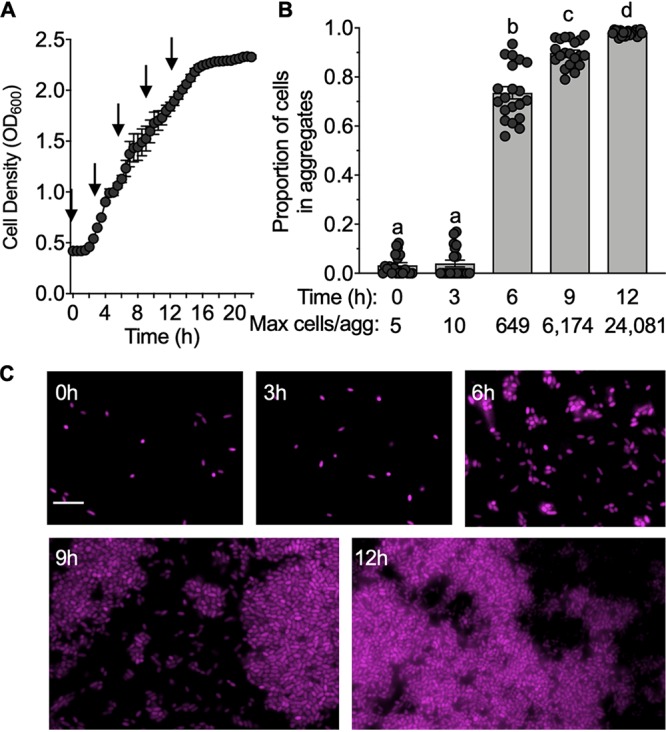
Aggregate formation occurs by 6 h. (A) Growth curve of RFP-tagged ES401 incubated in high-viscosity liquid medium for 22 h. Arrows indicate the time points when the cultures were examined for the presence of aggregates using fluorescence microscopy. Growth curves were performed three times, with the results of one representative experiment being shown (*n* = 4). (B) Proportion of cells from each time point that were associated with aggregates (three or more cells touching). Letters indicate significantly different values between time points (one-way ANOVA, *P < *0.001). The proportion of cells in aggregates was calculated for two experiments, and combined data are shown (*n* = 20). (C) Representative fluorescence microscopy images of ES401 from the growth curve shown in panel A. Bar = 5 μm. Error bars indicate standard errors.

To determine how quickly the cells formed aggregates, we grew ES401 in low-viscosity liquid to an OD_600_ of ∼0.5, transferred the cells to high-viscosity liquid, and monitored the cell density over 22 h (Fig. 5A). The culture was subsampled at the time points indicated in Fig. 5A and imaged to quantify the size and the proportion of cells within the aggregates, which we defined as clusters of three or more cells. At the beginning of the experiment and after 3 h, less than 5% of the cells were associated with small aggregates of less than 10 cells (Fig. 5B and C). By 6 h, ∼74% of the cells in the culture were in moderately sized aggregates of ∼650 cells (Fig. 5B). By 9 h, the proportion of cells associated with aggregates further increased to ∼90%, and >98% of cells were in aggregates by 12 h, with the maximum aggregate size reaching >24,000 cells per aggregate (Fig. 5B). These data indicate that, under the conditions used here, cells begin to aggregate at ∼3 to 6 h after the transition from low- to high-viscosity liquid. Given that inhibitor cells are primed for T6SS2-dependent killing after only 30 min in hydrogel (Fig. 3), these findings suggest that inhibitor cells activate T6SS2 prior to making contact with other cells in aggregates and, therefore, that T6SS2 activation is a direct result of a change in the physical environment, rather than a response to being in an aggregate.

### Competing genotypes coaggregate in hydrogel.

**FIG 6:**
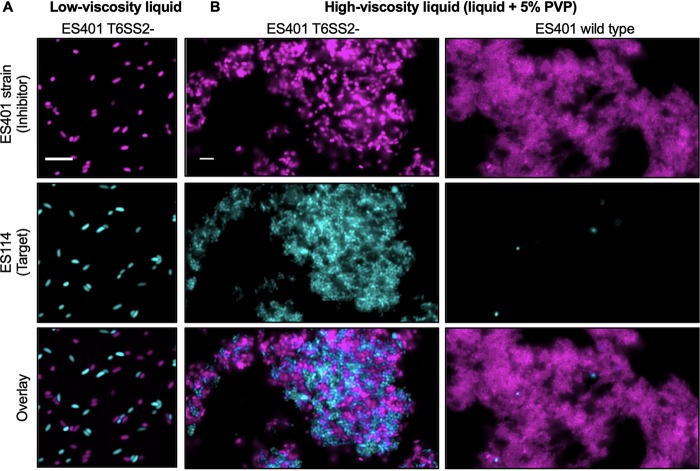
ES401 eliminates ES114 in mixed-strain aggregates. Fluorescence microscopy images of mixed cultures of GFP-tagged ES114 (cyan) and RFP-tagged wild-type or *vasA_2* mutant (T6SS2 mutant) ES401 (magenta) incubated in either low-viscosity liquid (A) or high-viscosity liquid (B) for 12 h. An overlay of the images of ES114 and ES401 is shown in the bottom row. Bars = 5 μm. Each experiment was performed twice with two biological replicates and five fields of view; one representative image is shown.

To determine whether multistrain aggregates form in hydrogel, mixed cultures of differentially tagged ES114 and ES401 *vasA_2* mutant strains were incubated in low- or high-viscosity liquid for 12 h. We chose to use the *vasA_2* mutant to observe cell-cell contact between inhibitor and target cells without the complication of ES114 being killed during the assay. In low-viscosity liquid, no aggregates were observed (Fig. 6A). In high-viscosity liquid, we observed coaggregates containing an equal percentage of each strain type: 50.6% ES114 and 49.4% ES401 *vasA_2* mutant (±7.4%, standard deviation) (Fig. 6B). Given that the target and T6SS2 mutant inhibitor cells were in contact with one another, we predicted that the target cells would be eliminated within aggregates when the inhibitor had a functional T6SS2. To test this prediction, we incubated ES114 and wild-type ES401 in high-viscosity liquid for 12 h and observed aggregates composed nearly exclusively of inhibitor cells, with only a few, rounded target cells being present (<0.28% of the population) (Fig. 6B). Taken together, these observations indicate that high-viscosity medium promotes mixed-strain aggregates where an inhibitor strain can eliminate target cells and further demonstrate the utility of hydrogel as a controlled, host-like liquid environment for studying bacterial aggregation and T6SS interactions.

### V. fischeri enhances T6SS and ribosomal protein expression in high viscosity.

**FIG 7:**
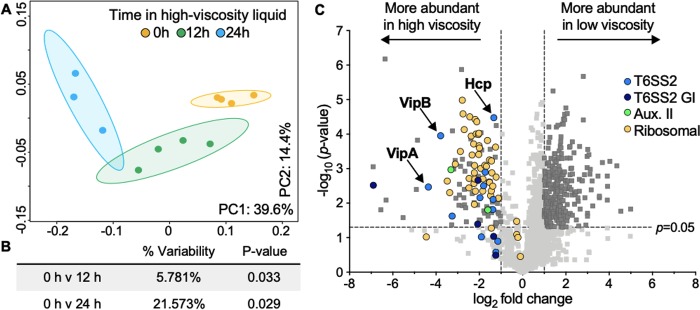
T6SS2 proteins are more abundant in high-viscosity liquid than in low-viscosity liquid. (A) Principal-coordinate analysis (PCoA) plots of proteomics data. The percentages on each axis indicate the amount of variation explained by the axis, and ellipses indicate 95% confidence intervals. (B) Adonis *P* values for pairwise comparisons between each treatment. (C) Volcano plot showing the log_2_ fold difference in protein abundance between 0 h and 24 h in high-viscosity treatments. Proteins with a negative log_2_ fold change value are more abundant in the high-viscosity treatment (left), and proteins with a positive log_2_ fold change value are more abundant in the low-viscosity treatment (right). Colors indicate the functional assignment of the proteins of interest: light blue, T6SS2 proteins; dark blue, proteins on the T6SS2 genomic island (GI); green, proteins encoded by T6SS auxiliary cluster II (Aux. II); yellow, ribosomal proteins. Data points above the dashed horizontal line had significant *P* values between treatments (Student's *t* test with the Bonferroni correction, *P < *0.05), and those outside the vertical dashed lines had a magnitude fold change of >|1| log_2_ between treatments (dark gray squares).

Given that V. fischeri undergoes significant physiological changes when transitioning between low- and high-viscosity conditions, we wanted to gain insight into how V. fischeri modulates protein expression in response to environmental viscosity. We quantified the proteome of ES401 grown in low-viscosity liquid, as well as that of ES401 after being transitioned to high viscosity for 1, 12, or 24 h (Fig. S3). Samples were collected in the same growth phase for each comparison to ensure that the differences in expression were due to the conditions tested (59). We detected 1,463 proteins (Table S1), or ∼38% of the proteins encoded by the ES401 genome (40), which is the portion of the proteome that we would expect to detect based on previous comprehensive proteomics studies (60). We first compared the proteomes of cultures in low- or high-viscosity liquid for 1 h (Fig. S3, black triangles) and found that only 14 proteins were significantly differentially expressed between treatments (analysis of variance [ANOVA], *P < *0.02) (Table S2), suggesting that 1 h in high viscosity is not sufficient to detect substantial changes in the proteome. We next compared the proteomes of cultures grown in low viscosity (0 h of exposure to high viscosity) to those of cultures exposed to hydrogel for 12 or 24 h (Fig. S3, gray triangles). We performed a principal-coordinate analysis (PCoA) of these proteomes, which revealed that the samples clustered by the duration of exposure to hydrogel (Fig. 7A). The 0- and 24-h treatments clustered the farthest from one another (Fig. 7A and B) (ANOVA, *P < *0.03), and the number of differentially abundant proteins (638 proteins) was significantly larger than that seen in the 0-h versus 12-h treatment comparison (53 proteins) (Tables S3 and S4).

10.1128/mBio.03060-19.4 FIG S3 Timeline for collection of proteomics samples. Cultures of strain ES401 were initially grown in low-viscosity liquid (gold) and transferred to fresh low- or high-viscosity medium (blue) every 12 h. Cultures were exposed to high-viscosity liquid for 0 h (top arrow), 12 h (middle arrow), or 24 h (bottom arrow). Triangles indicate when samples were collected for proteomics analysis, and comparisons were made between triangles of the same color. Experiments included four biological replicates that were run in parallel. Download FIG S3, DOCX file, 0.05 MB. Copyright © 2020 Speare et al. 2020 Speare et al. This content is distributed under the terms of the Creative Commons Attribution 4.0 International license.

10.1128/mBio.03060-19.5 TABLE S1 All proteins detected in the proteomes of ES401 incubated in high-viscosity (5% PVP) or low-viscosity (liquid) medium for 1 h, 12 h, or 24 h. NSAF values (in percent) represent relative protein abundances as a percentage of the total protein detected. Three to four replicates were included for each treatment. Download Table S1, XLSX file, 0.4 MB. Copyright © 2020 Speare et al. 2020 Speare et al. This content is distributed under the terms of the Creative Commons Attribution 4.0 International license.

10.1128/mBio.03060-19.6 TABLE S2 Proteins that were significantly more abundant after 1 h in either low- or high-viscosity medium treatments. NSAF values (in percent) represent relative protein abundances as a percentage of the total protein detected. *t* tests were performed for each protein, and Benjamini-Hochberg-corrected *P* values are shown. Four replicates were included for each treatment. Download Table S2, XLSX file, 0.01 MB. Copyright © 2020 Speare et al. 2020 Speare et al. This content is distributed under the terms of the Creative Commons Attribution 4.0 International license.

10.1128/mBio.03060-19.7 TABLE S3 Proteins that were significantly more abundant after 12 h in either low- or high-viscosity medium treatments. NSAF values (in percent) represent relative protein abundances as a percentage of the total protein detected. *t* tests were performed for each protein, and Benjamini-Hochberg-corrected *P* values are shown. Four replicates were included for each treatment. Download Table S3, XLSX file, 0.02 MB. Copyright © 2020 Speare et al. 2020 Speare et al. This content is distributed under the terms of the Creative Commons Attribution 4.0 International license.

To further analyze the 0-h versus 24-h exposure treatments, a volcano plot was generated to visualize the significantly differentially expressed proteins (Fig. 7C). Next, we identified all proteins encoded in the T6SS2 genomic island: both T6SS2 structural proteins (light blue) and other genomic island proteins (dark blue) were significantly more abundant under the high-viscosity condition than under the low-viscosity condition. Consistent with the increased *hcp_2* promoter reporter activity and the sheath assembly data, both Hcp and the VipA/VipB sheath proteins were more abundant in the high-viscosity liquid than in the low-viscosity liquid, as was one of the predicted T6SS effectors encoded in auxiliary cluster II. When we examined the other differentially expressed proteins, we noticed that 44 of the 60 predicted ribosomal proteins were significantly more abundant in high viscosity than in low viscosity (Fig. 7C; Table S4). It is therefore tempting to speculate that the corresponding increase of T6SS expression, which is a large nanostructure composed of many protein subunits and protein synthesis machinery, could be a mechanism for cells to support the building of T6SS sheaths, which is predicted to be energetically costly (61). Although this initial analysis revealed novel insights into how cells modulate T6SS and ribosomal protein expression, further analysis of the remaining differentially abundant proteins could reveal other physiological responses to changes in environmental viscosity. It is also notable that proteins encoded by genes belonging to the cellulose (62) and symbiosis polysaccharide (*syp*) biofilm gene clusters (63–65) were largely undetected, suggesting that aggregate formation in hydrogel likely occurs through an alternative mechanism.

10.1128/mBio.03060-19.8 TABLE S4 Proteins that were significantly more abundant after 24 h in either low- or high-viscosity medium treatments and that showed a greater than |1| log_2_ fold difference in protein abundance between treatments. NSAF values (in percent) represent relative protein abundances as a percentage of the total protein detected. *t* tests were performed for each protein, and Benjamini-Hochberg-corrected *P* values are shown (corrected *t* test). Category designations are included for proteins that are annotated as T6SS2 proteins, proteins encoded on the T6SS2 genomic island (T6SS2GI), and proteins in T6SS auxiliary cluster II (Aux II). Three to four replicates were included for each treatment. Download Table S4, XLSX file, 0.1 MB. Copyright © 2020 Speare et al. 2020 Speare et al. This content is distributed under the terms of the Creative Commons Attribution 4.0 International license.

### Conclusion.

**FIG 8:**
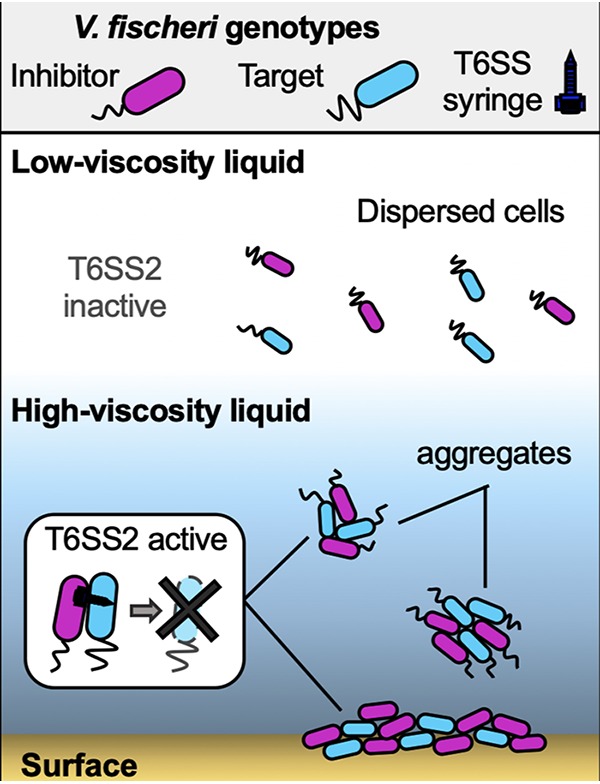
Conceptual model for the role of viscosity in modulating V. fischeri cell-cell contact and T6SS2 activity. In a low-viscosity, liquid environment, V. fischeri cells are physically dispersed and T6SS2 is functionally and transcriptionally inactive. High-viscosity medium facilitates cell-cell contact between different genotypes: high-viscosity liquid promotes the formation of mixed-strain aggregates by V. fischeri, while surfaces force cells into physical contact. High-viscosity and surface conditions promote increased T6SS2 expression, sheath assembly, and T6SS2-dependent killing.

Taken together, the findings of this work demonstrate that V. fischeri modulates the T6SS2-dependent killing of target cells in response to a transition from a lower-viscosity (i.e., planktonic) environment to the higher-viscosity and surface environments experienced in the host. Based on what is currently known about host colonization kinetics, the role of T6SS2 in preventing cocolonized crypts, and T6SS2 modulation described above, we propose a model for how T6SS2 may be activated by host-like viscosity to prime cells for interstrain competition for the host niche (Fig. 8). Juvenile Eupyrmna scolopes squid hatch without their light organ symbionts, which they acquire from the water column. Our data suggest V. fischeri cells in this planktonic state are physically dispersed, the levels of T6SS2 transcription and protein expression are low, and T6SS2 is functionally inactive. Planktonic V. fischeri cells aggregate in a mucus matrix on the light organ surface, where they experience a dramatic increase in viscosity (29, 65), which may act to prime cells for T6SS2-mediated competition. Founder cells then disperse from aggregates to enter the pores and travel through the ducts into the crypts, where populations proliferate. If a crypt is initially cocolonized by two competing strain types, direct contact between cells facilitates T6SS2-mediated interactions, resulting in crypts that are colonized exclusively by one strain type (2).

## MATERIALS AND METHODS

Media and growth conditions, strain and plasmid construction, coincubation assays, and single-cell fluorescence microscopy were performed as previously described (2, 66). β-Galactosidase assays were performed as previously described (44). See Text S1 in the supplemental material for additional experimental details.

10.1128/mBio.03060-19.1 TEXT S1 Supplemental information (SI) appendix. Download Text S1, DOCX file, 0.02 MB. Copyright © 2020 Speare et al. 2020 Speare et al. This content is distributed under the terms of the Creative Commons Attribution 4.0 International license.

### Quantifying the viscosity of the media.

Medium viscosity was determined by quantifying the velocity of a small glass sphere as it traveled through a graduated cylinder containing PVP-amended liquid medium. The viscosity of the medium (in grams centimeter^−1^ second^−1^) was calculated using the following equation: viscosity = {2[(sphere density) − (medium density)] × gravity × (sphere radius)^2^}/[9 × sphere velocity], where the sphere and medium densities are in grams centimeter^−3^, the gravity is in centimeters second^−2^, the sphere velocities are in centimeters per second, and the sphere radius is in centimeters (67). This experiment was performed twice with 10 replicates per experiment.

### Quantification of aggregate size and proportion of strain types.

Aggregates were visualized using cultures of V. fischeri strains harboring either pVSV102 (GFP) or pVSV208 (dsRed red fluorescent protein [RFP]) and an Olympus BX51 microscope outfitted with an Hamamatsu C8484-03G01 camera. Cultures were initially grown in low-viscosity liquid, normalized to an OD_600_ of 1.0, and then 10 μl was transferred into high-viscosity liquid. The cultures were then incubated for up to 12 h, after which 5 μl of each culture was spotted onto a glass slide and imaged within 30 min using a 60× (numerical aperture, 1.30) oil Ph3 objective lens. Images were captured using MetaMorph software. The proportion of aggregated cells was determined by using the image/adjust/threshold command to segment the image into particle and background components using ImageJ software. The number of particles and the area of each particle were calculated, and then the number of cells per particle was determined by dividing the particle size by the average size of a V. fischeri cell (1.5 μm^2^). Particles containing three or more cells were defined as aggregates. For experiments with mixed-strain aggregates, the proportion of strain types within each aggregate was determined by first calculating the total area of an aggregate in the composite image of both strains. Next, the proportion of the aggregate area that was either GFP or dsRed was determined and divided by the total area of the aggregate. These values were compared to ensure that they added up to 1.0 (the entire area of the aggregate) and to get the final ratio.

### Sample preparation for proteomics.

Cultures of strain ES401 were grown in standing cultures at 24°C in either low- or high-viscosity liquid and subcultured every 12 h into fresh medium (Fig. S3). Samples were incubated in high-viscosity liquid for either 0, 1, 12, or 24 h and collected by centrifugation at the time points indicated above, and cell pellets were frozen at −80°C. Thirty microliters of SDT-lysis buffer (4% [wt/vol] SDS, 100 mM Tris-HCl, 0.1 M dithiothreitol) was added to each cell pellet, and then the samples were incubated at 95°C for 10 min for cell lysis. Tryptic digests of protein extracts were prepared following the filter-aided sample preparation (FASP) protocol (68). In addition to the minor modifications described by Kleiner et al. (69), lysate was not cleared by centrifugation after boiling the sample in lysis buffer. We loaded the whole lysate onto the filter units used for the FASP procedure. Centrifugation times were reduced to 20 min, which is different from the centrifugation time used by Kleiner et al. (69). Peptide concentrations were determined with a Pierce micro-bicinchoninic acid assay (Thermo Fisher Scientific), using an Epoch2 microplate reader (BioTek), following the manufacturer’s instructions.

### LC-MS/MS.

All samples were analyzed by one-dimensional liquid chromatography-tandem mass spectrometry (LC-MS/MS) as described previously (70), with the modification that a 75-cm analytical column and a 140-min-long gradient were used. For each sample run, 400 ng peptide was loaded with an UltiMate 3000 RSLCnano liquid chromatograph (Thermo Fisher Scientific) in loading solvent A (2% acetonitrile, 0.05% trifluoroacetic acid) onto a 5-mm, 300-μm (inside diameter) C_18_ Acclaim PepMap100 precolumn (Thermo Fisher Scientific). Separation of the peptides on the analytical column (75-cm by 75-μm analytical Easy-Spray column packed with PepMap RSLC C_18_ [particle size, 2 μm] material; Thermo Fisher Scientific) was achieved at a flow rate of 300 nl min^−1^ using a 140-min gradient going from 95% buffer A (0.1% formic acid) to 31% buffer B (0.1% formic acid, 80% acetonitrile) in 102 min and then to 50% buffer B in 18 min to 99% buffer B in 1 min and ending with 99% buffer B. The analytical column was heated to 60°C and was connected to a Q Exactive HF hybrid quadrupole-Orbitrap mass spectrometer (Thermo Fisher Scientific) via an Easy-Spray source. Eluting peptides were ionized via electrospray ionization (ESI). Carryover was reduced by one wash run (injection of 20 μl acetonitrile, 99% buffer B) between samples. Full scans were acquired in the Orbitrap mass spectrometer at a resolution of 60,000. The 15 most abundant precursor ions were selected for fragmentation, and MS/MS scans were acquired at a resolution of 15,000. A mass (*m/z*) of 445.12003 was used as the lock mass. Ions with a charge state of +1 were excluded from the MS/MS analysis. Dynamic exclusion was set to 18 s. Roughly 135,000 MS/MS spectra were acquired per sample.

### Protein identification and statistical analysis.

A database containing protein sequences from V. fischeri ES401 (GenBank accession number SRJG00000000.1) downloaded from NCBI (https://www.ncbi.nlm.nih.gov/Traces/wgs/SRJG01) was used. Sequences of common laboratory contaminants were included by appending the cRAP protein sequence database (http://www.thegpm.org/crap/). The final database contained 3,925 protein sequences. Searches of the MS/MS spectra against this database were performed with the Sequest HT node in Proteome Discoverer software (version 2.2.0.388; Thermo Fisher Scientific), as described by Petersen et al. (71). Only proteins identified with medium or high confidence were retained, resulting in an overall false discovery rate of <5%. For protein quantification, normalized spectral abundance factors (NSAFs) (72) were calculated and multiplied by 100 to obtain relative protein abundance (in percent).

Principal-coordinate analysis (PCoA) was performed using a Bray-Curtis-based dissimilarity matrix from the vegan package in R (73). A contingency table was generated by comparing average protein abundance between treatments using a Student's *t* test, corrected for multiple comparisons using the Benjamini-Hochberg procedure (74). A volcano plot was generated by graphing the negative log_10_ *P* value and log_2_ fold change between treatments. NSAFs of 0 were replaced by the limit-of-detection value of 0.000189 to allow for comparisons of the log_2_ fold change between treatments.

### Data availability.

The mass spectrometry metaproteomics data and protein sequence database have been deposited in the ProteomeXchange Consortium via the PRIDE (75) partner repository with the data set identifier PXD015403.
