# Supplementary material for: Environmental Viscosity Modulates Interbacterial Killing during Habitat Transition
Source: mBio. 2020 Feb 4;11(1):e03060-19. doi: 10.1128/mBio.03060-19 (PMC7002345; doi:10.1128/mBio.03060-19)
Supplement: FIG S1 [file mBio.03060-19-sf001.docx]

**Figure S1. *V. fischeri* do not use PVP as a sole carbon source.** Growth yields of clonal cultures of strains ES114 (cyan) and ES401 (magenta) grown for 12 h in minimal media supplemented with 10 mM N-Acetylglucosamine (NAG) or 5% PVP. Experiments were performed three times and combined data are shown (n=12). Treatments that do not share letters denote statistical difference (Student’s t-test: *P*<0.001).
