# Supplementary material for: Environmental Viscosity Modulates Interbacterial Killing during Habitat Transition
Source: mBio. 2020 Feb 4;11(1):e03060-19. doi: 10.1128/mBio.03060-19 (PMC7002345; doi:10.1128/mBio.03060-19)
Supplement: FIG S2 [file mBio.03060-19-sf002.docx]

**Figure S2. T6SS2 activation is independent of cell density and key quorum sensing genes.** (A) Cultures of ES401 harboring VipA_2-GFP were grown in liquid LBS supplemented with 0.5 mM IPTG to an OD_600_ 0.5 - 2.0 and imaged immediately. (B) Luminescence curve showing specific luminescence (RLU/OD_600_) for ES401 wild type (WT), *vasA_2* mutant*, luxU* mutant, and *luxR* mutant strains in high-viscosity liquid media (5% PVP). (C) CFUs for coincubation assay between ES114 (cyan) and ES401 (magenta) strains that were incubated in high-viscosity liquid media for 12 hours. Asterisks indicate *P<*0.001 for a Student’s t-test comparing the CFUs of ES114 with ES401 at 12 hours. Error bars indicate standard error. All experiments were performed either twice with two biological replicates and five fields of view for each replicate (A) or three times with four biological replicates (B and C); combined data are shown (A, n=20; B and C, n=12).
