# Supplementary material for: Environmental Viscosity Modulates Interbacterial Killing during Habitat Transition
Source: mBio. 2020 Feb 4;11(1):e03060-19. doi: 10.1128/mBio.03060-19 (PMC7002345; doi:10.1128/mBio.03060-19)
Supplement: FIG S3 [file mBio.03060-19-sf003.docx]

**Figure S3. Timeline for collection of proteomics samples.** Cultures of ES401 were initially grown in low-viscosity liquid (gold) and transferred to fresh low- or high-viscosity media (blue) every 12 h. Cultures were exposed to high-viscosity liquid for 0 h (top arrow), 12 h (middle arrow), or 24 h (bottom arrow). Triangles indicate when samples were collected for proteomics analysis, and comparisons were made between triangles of the same color. Experiments included four biological replicates that were run in parallel.
