# Supplementary material for: Environmental Viscosity Modulates Interbacterial Killing during Habitat Transition
Source: mBio. 2020 Feb 4;11(1):e03060-19. doi: 10.1128/mBio.03060-19 (PMC7002345; doi:10.1128/mBio.03060-19)
Supplement: TEXT S1 [file mBio.03060-19-s0001.docx]

***SI Appendix***

**Media and growth conditions.** *V. fischeri* strains were grown in LBS medium or Tris Minimal Media (MM) (1) at 24°C, the average temperature of sea water from Kaneohe Bay, and *E. coli* strains were grown in either LB medium or Brain Heart Infusion (Difco) at 37°C. Antibiotic selection for *V. fischeri* and *E. coli* strains, and plasmid maintenance were performed as described previously (2-4). High-viscosity, liquid medium was made by supplementing LBS broth with 5% polyvinylpyrrolidone (PVP) weight/volume unless otherwise indicated. Growth and luminescence curves were performed using a Tecan Plate reader as described previously (5).

**Strain and plasmid construction.** *V. fischeri* strains ES114 (6) and ES401 (7) were isolated from *Eupryma scolopes* light organs. To construct mutants in *V. fischeri* strain ES401, mutant alleles were mobilized on plasmids into wild-type ES401 by triparental mating using CC118λpir pEVS104 (2) as a conjugative helper, and then screening for appropriate antibiotic resistance and verified using PCR. The ES401 *vasA_2* (*tssF*) mutant strain (ANS2100) was constructed by moving pAS2038 (5) into wild-type ES401. The ES401 *luxU* mutant strain (LAS018) was constructed by moving pHK79 (8) into wild-type ES401. To construct the *luxR* disruption mutant, approximately 0.5 kb of the *luxR* gene was PCR amplified using primers LS026 (GCGAATTCGAGCTCGGTACCTACTAGAATATGACCCTGTAGTCG) and LS027 (GACTCTAGAGGATCCCCGGGGTATGGACAATTAATGGCGCC) from ES401 gDNA. The resulting PCR product was cloned into the KpnI and SmaI sites of plasmid pEVS122, resulting in the *luxR* disruption construct, pLS08. The *luxR* disruption construct on pLS08 was moved into strain ES401, resulting in strain LAS020. To construct the P*_hcp_2_*-*lacZ* fusion promotor reporter plasmid (pAG01) the 465 bp upstream of *hcp_2* (*tssD*) and first 13 bp of the coding sequence was PCR amplified using primers AG01b (ATCCTAGGAGTTAGTTTCAATCATTAGCCAGAG) and AG02 (TAGCTAGCGCTGGAGTTGGCATGCTATTATC) from *V. fischeri* ES12 gDNA. The resulting PCR product was cloned into AvrII and NheI sites in pAKD701 (9). Strains were differentially tagged for coincubation assays and single-cell fluorescence microscopy with either pVSV102 (Kn^R^, GFP+) or pVSV208 (Cm^R,^ dsRed+) (10).

**Coincubation Assay.** Coincubation assays were performed as described previously (2, 11). Cultures of *V. fischeri* strains grown in LBS broth supplemented with the appropriate antibiotic at 24°C were diluted to an OD_600_ of 1.0, mixed in a 1:1 ratio based on OD, 10 µl of the mixture was spotted into wells containing 1 mL of low- or high-viscosity liquid or onto LBS agar plates and incubated at 24°C without shaking. At indicated time points, strains in each coincubation were quantified by plating serial dilutions onto LBS plates supplemented with antibiotics selective for each strain.

**β-Galactosidase Assay of promoter activity.** *V. fischeri* strains containing *lacZ*-based reporter plasmids were grown overnight in low- or high-viscosity liquid LBS media containing the appropriate antibiotic. Cultures were diluted 1000-fold into media of the same type containing the appropriate antibiotic and grown for 12 h (Fig 2B) or to an OD_600_ of ~0.55 (+/- 0.10) (Fig 2C). Cells were harvested by pelleting 0.9 mL of culture. Supernatant was discarded and cells were frozen at -20°C for no longer than 24 h. Cell pellets were resuspended in 0.9 mL Z buffer and β-galactosidase assay performed using a modified Miller assay as previously described (12).

**Single-cell Fluorescence Microscopy.** Single-cell fluorescence microscopy was performed by visualizing cultures of ES401 carrying the IPTG-inducible VipA_2-GFP (TssC) expression vector (pSNS119) (5). Cultures were grown overnight in the specified media supplemented with kanamycin and 0.5 mM isopropyl-β-D-1-thiogalactopyranoside (IPTG), then diluted 1:100 into fresh media with the same supplements, grown to an OD ~1.5, and imaged using an Olympus BX51 microscope outfitted with a Hammatsu C8484-03G01 camera and a 100X/1.30 Oil Ph3 objective lens as described previously (5).

**References**

1. Visick KL, Hodge-Hanson KM, Tischler AH, Bennett AK, Mastrodomenico V. 2018. Tools for rapid genetic engineering of *Vibrio fischeri*. Applied Environmental Microbiology 84:e00850-18.

2. Dunn AK, Martin MO, Stabb EV. 2005. Characterization of pES213, a small mobilizable plasmid from *Vibrio fischeri*. Plasmid 54:114-134.

3. Herrero M, de Lorenzo V, Timmis KN. 1990. Transposon vectors containing non-antibiotic resistance selection markers for cloning and stable chromosomal insertion of foreign genes in gram-negative bacteria. Journal of Bacteriology 172:6557-6567.

4. Hanahan D. 1983. Studies on transformation of *Escherichia coli* with plasmids. Journal of molecular biology 166:557-580.

5. Speare L, Cecere AG, Guckes KR, Smith S, Wollenberg MS, Mandel MJ, Miyashiro T, Septer AN. 2018. Bacterial symbionts use a type VI secretion system to eliminate competitors in their natural host. Proc Natl Acad Sci U S A 115:E8528-E8537.

6. Boettcher K, Ruby E. 1990. Depressed light emission by symbiotic *Vibrio fischeri* of the sepiolid squid *Euprymna scolopes*. Journal of Bacteriology 172:3701-3706.

7. Lee K-H, Ruby EG. 1994. Effect of the squid host on the abundance and distribution of symbiotic *Vibrio fischeri* in nature. Applied Environmental Microbiology 60:1565-1571.

8. Kimbrough JH, Stabb EV. 2016. Antisocial luxO mutants provide a stationary-phase survival advantage in Vibrio fischeri ES114. Journal of Bacteriology 198:673-687.

9. Dunn AK, Stabb EV. 2008. Genetic analysis of trimethylamine N-oxide reductases in the light organ symbiont *Vibrio fischeri* ES114. Journal of Bacteriology 190:5814-5823.

10. Dunn AK, Millikan DS, Adin DM, Bose JL, Stabb EV. 2006. New rfp-and pES213-derived tools for analyzing symbiotic *Vibrio fischeri* reveal patterns of infection and lux expression in situ. Applied Environmental Microbiology 72:802-810.

11. Speare L, Septer AN. 2019. Coincubation Assay for Quantifying Competitive Interactions between Vibrio fischeri Isolates. Journal of visualized experiments: JoVE.

12. Bose JL, Rosenberg CS, Stabb EV. 2008. Effects of luxCDABEG induction in *Vibrio fischeri:* enhancement of symbiotic colonization and conditional attenuation of growth in culture. Archives of Microbiology 190:169.
